# Randomized, Placebo‐Controlled Pilot Study of Naproxen During Dental Implant Osseointegration

**DOI:** 10.1002/cre2.70065

**Published:** 2025-01-05

**Authors:** Hattanas Kumchai, Daniel I. Taub, Ryan E. Tomlinson

**Affiliations:** ^1^ Department of Orthopaedic Surgery Thomas Jefferson University Philadelphia Pennsylvania USA; ^2^ Department of Oral and Maxillofacial Surgery Thomas Jefferson University Philadelphia Pennsylvania USA

**Keywords:** dental implants, implant stability, implant surgery, ISQ, NSAIDs, osseointegration

## Abstract

**Objectives:**

Nonsteroidal anti‐inflammatory drugs (NSAIDs) are often prescribed following the placement of dental implants, but the effects of these drugs on the osseointegration process are poorly understood. We designed a randomized, placebo‐controlled pilot study to quantitatively assess the effect of NSAIDs during early implant osseointegration.

**Materials and Methods:**

Subjects receiving a maxillary dental implant were randomized to take naproxen or placebo for 7 days after the surgery. Implant osseointegration was quantified using Resonance Frequency Analysis device. Implant‐Stability‐Quotient (ISQ) measurement was performed at the time of surgery and at follow‐up visits 1, 4, and 16 weeks after surgery. Periapical radiographs were taken to measure the marginal bone level. Separately, a questionnaire of NSAIDs usage was provided to subjects presenting with early implant failure.

**Results:**

After 4 weeks, ISQ values increased modestly ( + 1%) in subjects receiving naproxen whereas subjects receiving placebo had a much larger increase in ISQ value (+41%). We observed 55% more marginal bone loss at 4 weeks, and 52% at 16 weeks in the naproxen group compared to the placebo group. These results were not found to have statistically significant between groups (*p* ≥ 0.05). These effect sizes and variance were used to conduct a power analysis to determine the necessary sample size for future studies. Furthermore, our separate questionnaire study revealed that 68% of our patients with early failed dental implants reported a history of NSAIDs usage after the surgery.

**Conclusion:**

In conclusion, this pilot study provides effect sizes and sample size estimates for future studies to definitively determine recommendations regarding NSAID usage following dental implant surgery. Nonetheless, our study did not observe any statistically significant differences in ISQ value or marginal bone loss after up to 16 weeks of follow‐up between subjects from naproxen and placebo groups.

## 1 Introduction

The use of dental implants is the gold standard to restore edentulous sites. The success of this procedure is entirely dependent on osseointegration, which is the process of bone forming a structural and functional connection with the implant (Brånemark et al. 1969; Adell et al. 1970). Although dental implants generally have excellent long‐term survival rates, implant failure in the first few months due to incomplete osseointegration occurs in about 2% of patients (Chrcanovic, Albrektsson, and Wennerberg 2014).

Following dental implant surgery, nonsteroidal anti‐inflammatory drugs (NSAIDs) may be recommended for the relief of pain and swelling. NSAIDs prevent the synthesis of prostaglandin E2 (PGE2) by inhibiting the cyclooxygenase (COX) enzymes, COX1 and COX2 (Rainsford 2007; Mitchell et al. 1993). However, PGE2 is part of an inflammatory signaling pathway that is critically important for bone healing and repair. The consensus from a large body of literature is that NSAIDs usage is deleterious for fracture repair of long bones (Geusens et al. 2013; Al Farii et al. 2021; Lam 2014; Brown et al. 2004). However, animal studies have illustrated that these effects strongly depend on the timing, dose, and duration of NSAIDs treatment (Geusens et al. 2013). Furthermore, the harmful effects of NSAIDs may be exacerbated in the repair of craniofacial bones, which heal poorly as compared to similar‐sized defects in long bones (Mouraret et al. 2014). For example, mice lacking COX2 displayed robust non‐union of a calvarial fracture that healed normally in wild‐type mice (Chikazu et al. 2011). Furthermore, a recent retrospective analysis of failed dental implants found that perioperative NSAIDs usage was associated with 3.2 times more cases of radiographic bone loss and 1.9 times more cases of cluster failure (> 50% of implants placed) (Winnett et al. 2016). Similarly, a systematic review of osseointegration literature that reported NSAIDs use data (comprising 20 in vivo and 9 in vitro studies) concluded that COX2 inhibitors may impair the osseointegration process during the Postoperative period (Gomes et al. 2015). Nonetheless, despite potential contraindications, NSAIDs are considered safe and effective treatment for a variety of painful conditions in humans, including Postoperative pain due to orthopedic procedures (Ong et al. 2007), and are often recommended to patients for pain relief to avoid prescription narcotics (Manchikanti et al. 2012).

Therefore, the goal of this pilot study was to evaluate if implant stability, as quantified by either resonance frequency analysis or marginal bone loss, is affected by the use of NSAIDs during dental implant osseointegration. We hypothesized that the use of NSAIDs in the immediate postoperative period would lead to decreased implant stability due to impaired dental implant osseointegration.

## 2 Materials and Methods

**Figure 1:**
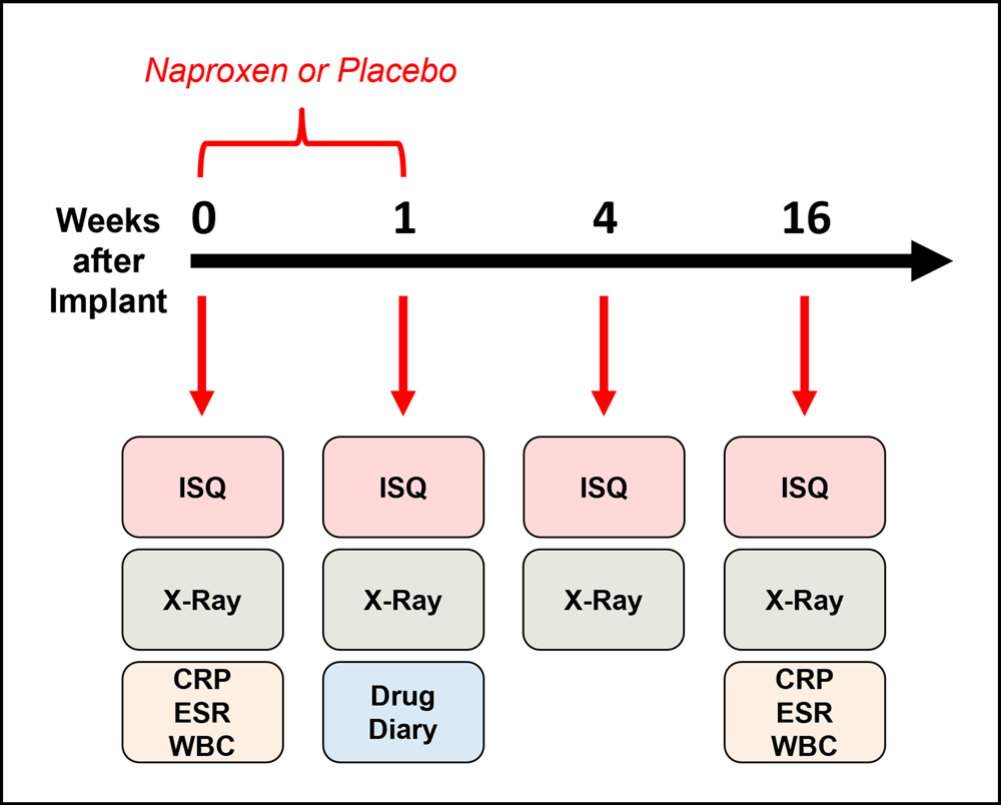
Overall timeline of the study. Subjects enrolled in the study received naproxen or placebo in the first week of the study. Implant stability was assessed by ISQ and X‐ray at implantation and three follow‐ups (1, 4, 16 weeks). CRP, ESR, and WBC were used to rule out infection at implantation and last follow‐up.

This randomized controlled clinical trial was by the Institutional Review Board (IRB) of Thomas Jefferson University (IRB #17 G.451) including ethical approval and protection of human subjects. The overview timeline of the study is illustrated in Figure 1.

### 2.1 Subjects

Subjects eligible for surgical implant placement at the Department of Oral and Maxillofacial Surgery, Thomas Jefferson University Hospitals, were invited to participate in the study. The inclusion criteria were (1) demonstrated need for maxillary implant, (2) provision of informed consent, (3) 25 to 76 years old, (4) edentulism at implant site for at least 30 days, (5) presence of opposing teeth or prostheses to the implant site, and (6) judged by the investigator as likely to achieve adequate initial implant stability upon placement. Exclusion criteria were (1) current smoking, (2) uncontrolled diabetes mellitus, (3) alcohol or drug abuse, (4) chronic NSAIDs use, (5) history of corticosteroid use within 1 year, (6) history of chemotherapy or radiation therapy within 5 years, (7) history of metabolic bone disease within 5 years, (8) allergy or aversion to Ibuprofen or Tylenol #3, (9) disease or condition that could compromise osseointegration as determined by the investigator, (10) judged by the investigator to be unlikely to comply with follow‐up procedures, and (11) simultaneous participation in another clinical study. The subjects were enrolled in the study after written consent was obtained. All patients were deemed to be periodontally stable before implant placement.

### 2.2 Surgical Protocol

All implant surgeries were conducted under local anesthesia by the same clinician. After initial incisions, full‐thickness flaps were raised. Subsequently, the implants were placed according to the manufacturer's instructions. The osteotomy was performed occasionally using a surgical stent. After achieving the primary stability of the implant, each subject then had a baseline Implant Stability Quotient (ISQ) measurement performed twice by using a Resonance Frequency Analysis device (Osstell IDx, Osstell, Sweden) in the buccolingual and mesiodistal directions. The lower ISQ number for each time point was selected for analysis. Then, a healing abutment was placed for transmucosal healing. A standard periapical radiograph of the implant was then taken. Primary closure around the healing abutment was obtained with sutures. Blood collection was also performed for measurements of C‐reactive protein (CRP), erythrocyte sedimentation rate (ESR), and white blood cells (WBC) to rule out low‐grade infection.

### 2.3 Postoperative Management and Data Collection

Following surgery, each subject was randomized (simple blinded randomization) to receive either a 7‐day supply of naproxen (220 milligram every 8 h; 21 tablets total) or placebo, then instructed to take it for the first 7 days after surgery. Both groups also receive acetaminophen 500 mg every 6 h for 7 days. In addition, acetaminophen‐codeine (Tylenol #3, 300 mg‐30 mg every 6 h as needed for 3 days) was provided as a rescue medication for both groups. A study diary was provided to each subject as a log to document their use of the study and rescue medications as well as a quantitative rating of their pain and swelling level for the first 7 days after the surgery. Subjects were prescribed antibiotics for 5 days after the surgery (amoxicillin 500 mg every 8 h, or clindamycin 300 mg every 8 h for patients who have an allergic history to amoxicillin). Subjects were scheduled for follow‐up visits 1, 4, and 16 weeks after surgery. ISQ measurement was performed at every follow‐up visit by the same clinician who performed the surgery. Standard periapical radiograph was also taken. Blood collection was also performed at the final follow‐up (16 weeks) for remeasurement of CRP, ESR, and WBC.

### 2.4 Radiographic Analysis

The marginal bone level was defined as the maximum distance from the implant‐abutment interface on the implant side to the marginal bone (Kim et al. 2017). The marginal bone level was measured from the mesial and distal sides of an implant in millimeters using CLINIVIEW 11 (Dexis, Pennsylvania, USA). Only the vertical marginal bone level was measured. Two clinicians recorded the mesial and distal aspects of each implant and intraclass correlation coefficient was calculated.

### 2.5 History of NSAIDs Usage in Subjects With a Failed Dental Implant

Separately, we received approval from the Institutional Review Board of Thomas Jefferson University to recruit subjects with early implant failure requiring explantation for a subject questionnaire (IRB #17 G.448). Here, eligible subjects were recruited and enrolled at the Department of Oral & Maxillofacial Surgery, Thomas Jefferson University Hospitals. The subjects were provided a questionnaire to document postoperative NSAIDs usage. The questionnaire was based on the National Health And Nutrition Examination Survey (NHANES) (Davis et al. 2017).

### 2.6 Statistical Analysis

Statistical analysis was carried out using GraphPad Prism (version 9.0.0, GraphPad Software Inc., San Diego, CA). Demographic data, implant information (implant location, height, and diameter), and reported rescue medication usage were compared between groups using the chi‐square test. ISQ data and marginal bone levels were analyzed using a two‐way ANOVA. A calculated *p*‐value less than 0.05 was considered statistically significant. Finally, we estimated required sample sizes using a power analysis at 80% and 90% power at the 0.05 level of significance for both ISQ and marginal bone levels.

## 3 Results

### 3.1 Subjects and Implants

Twelve subjects were recruited for this study. Seven subjects were in NSAIDs group, while five subjects were in the placebo group (control). Detailed subject demographics and implant characteristics are provided in Table 1. There were no significant differences in sex, implant location, implant height, implant diameter, or implant system between groups. CRP, ESR, and WBC from the blood collection of the subjects were all below the predetermined cutoff values (14 mg/L, 67 mm/h, and 14 × 10^9^ L^−1^, respectively). One subject enrolled in the naproxen group was removed from the study due to a failed implant uncovered at 1‐week follow‐up.

**Table 1:** Demographics and clinical parameters of study population and implant sites.

	Naproxen		Placebo		Sig
	*n*	%	*n*	%	
Subjects	7	58.3	5	41.7	
Age (y)	57.14		62.6		
Sex					NS
Male	6	85.7	3	60	
Female	1	14.3	2	40	
Implant location					NS
Anterior	1	14.3	1	20	
Premolar	4	57.1	2	40	
Molar	2	28.6	2	40	
Implant length					NS
8 to < 10 mm	0	0	2	40	
10 mm	4	57.1	2	40	
> 10–12 mm	3	42.8	1	20	
Implant diameter					NS
3–4 mm	3	42.9	1	20	
> 4 mm	4	57.1	4	80	
Implant system					NS
Nobel Biocare	2	28.6	0	0	
Straumann	0	0	2	40	
Astra	3	42.8	2	40	
Dentsply	1	14.3	1	20	

### 3.2 Self‐Reported Subjective Assessments of Pain and Swelling

**Figure 2:**
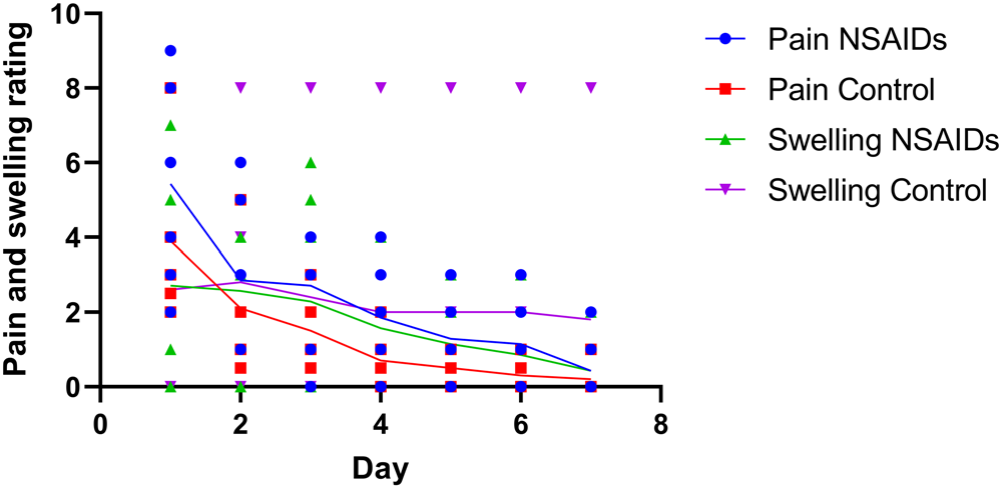
Subject pain and swelling. Pain and swelling were self‐reported on a 10‐point scale by subjects in control and NSAIDs group during first week following implant surgery. Individual data points as well as trendlines are shown for both metrics. There were no significant differences between groups.

Each subject self‐reported their pain and swelling on a numeric scale for the first week after implant surgery (Figure 2). Subjects in both groups reported more pain on the first day after surgery than on the last 3 days. Although there was no significant difference in self‐reported pain level between groups, a significantly higher percentage of patients in the placebo group (80%) required rescue medication as compared to NSAIDs group (14.3%). There was also no significant difference in reported swelling ratings between groups.

### 3.3 Clinical and Radiographic Assessments

**Figure 3:**
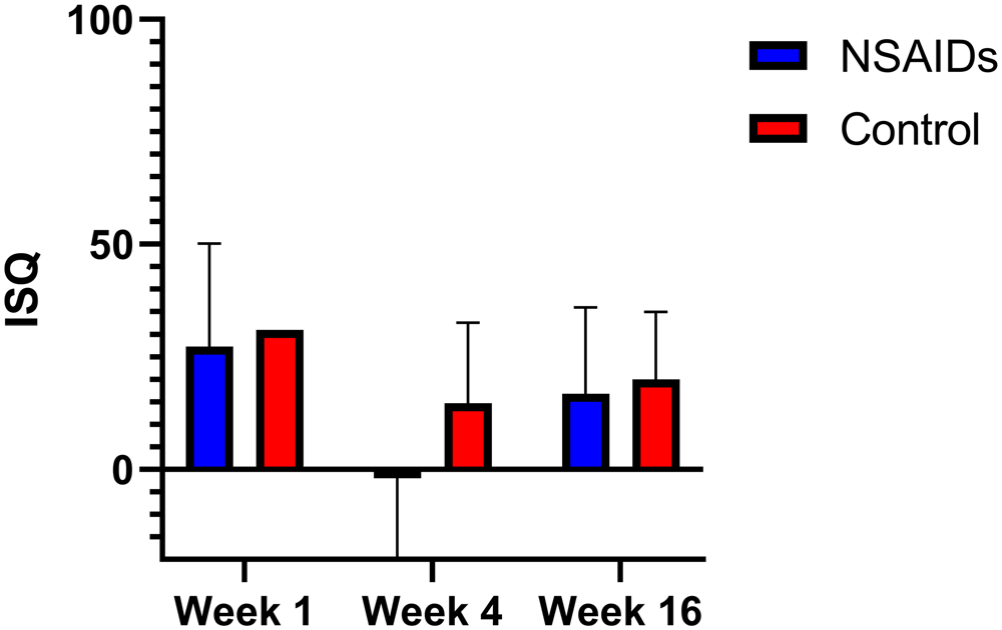
Implant stability by ISQ. Implant stability quotient (ISQ) was quantified at 1, 4, and 16 weeks following implant surgery in both the control and NSAIDs groups. There were no significant differences between groups at any time point.

**Figure 4:**
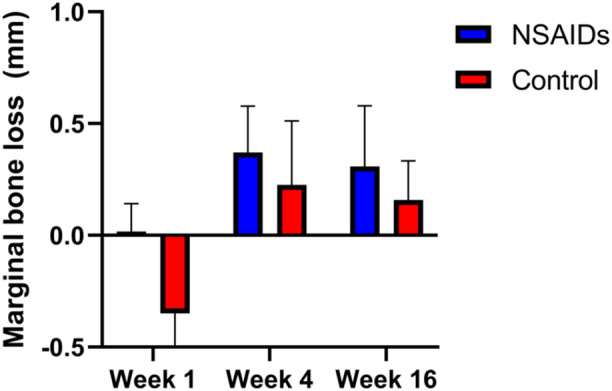
Marginal bone loss. Standard X‐ray was used to quantify marginal bone loss in subjects in NSAIDs and control group at 1, 4, and 16 weeks following implant surgery. There were no significant differences between groups at any time point.

ISQ values of each group compared to baseline at the time of implant surgery are shown in Figure 3. Here, we observed no significant increases in ISQ values at 4 weeks compared to baseline in subjects receiving naproxen, whereas subjects receiving placebo had an increased ISQ ( +41%), although this difference was not statistically significant. Similarly, we observed a smaller increase in ISQ values in the naproxen group ( +34%) compared to the placebo group ( +67%) at 16 weeks, although this difference was not statistically significant between groups. Similarly, we observed no statistically significant difference in marginal bone loss between groups at any time point (Figure 4). The intraclass correlation coefficient of measured marginal bone loss between two clinicians was 0.72.

### 3.4 Sample Size Calculation

Mean ISQ and marginal bone loss value from both groups at all time points were used to determine the sample size required to achieve 80% and 90% statistical power (Table 2). Our data indicates that future work using mean ISQ to determine the effect NSAIDs on dental implant osseointegration would require a minimum sample size of 64 subjects. In contrast, our data suggests that a study utilizing marginal bone loss may be sufficiently powered with a sample size as low as 7–10 subjects.

**Table 2:** Sample size (*n*) for each group required to achieve adequate statistical power at 80% and 90% power at each time point.

	Sample size (*n*)		
	Power		
	80%	90%	Range
ISQ			
1 week	303	405	303–405
4 weeks	64	86	64–86
16 weeks	431	577	431–577
Total	163	217	163–217
Marginal bone loss			
1 week	7	10	7–10
4 weeks	56	77	56–77
16 weeks	39	52	39–52
Total	43	57	43–57

### 3.5 History of NSAIDs Usage in Subjects With a Failed Dental Implant

Separately, 18 additional subjects presenting to our clinic with early dental implant failure were recruited to provide information regarding their current NSAID usage as well as NSAIDs usage immediately following implant surgery. Here, subjects were 38% male and had an average age of 56.2 (range: 29–91). We observed that 67.7% (12 of 18) of the subjects with failed dental implants reported a history of NSAIDs usage after the surgery. Within the subject groups that took NSAIDs, 91.7% (11 of 12) of subjects provided a history of nonaspirin NSAIDs use, while 9.5% (2 of 12) provided a history of aspirin use.

## 4 Discussion

Despite strong evidence that PGE2 synthesis through the action of COX2 is a critical component of bone repair, the effect of NSAIDs on dental implant osseointegration is poorly understood. Here, we did not observe statistically significant effects on implant stability due to a standard dose of naproxen for a week immediately following dental implant surgery. Nonetheless, naproxen usage was associated with a significant decrease in the use of rescue medication following surgery, suggesting relief of inflammation, pain, and swelling associated with the operation. Furthermore, our pilot data provide new insights regarding the effect size of two complementary and orthogonal measures that can be used to quantify implant stability in this scenario. As a result, our study indicates that the use of marginal bone loss may be sufficient to definitively quantify the negative effects of NSAID usage on osseointegration following dental implant surgery.

Primary stability of the dental implant, as determined immediately after the insertion of the implant into the osteotomy site (Javed et al. 2013), mainly depends on bone‐to‐implant contact. Bone quality, implant length, and diameter are all influential on the primary implant stability (Bilhan et al. 2010). In this study, we assessed the primary stability of implants by ISQ value from resonance frequency analysis. A study from Scarano et al. (2007). suggested that implants with an ISQ < 40 are irretrievably lost. Importantly, all implants in this study had an ISQ value above 40 by the end of the study period (16 weeks). Interestingly, we observed one failed implant at the 1‐week follow‐up in a subject in the naproxen group. Although dental implant failure is multifactorial (Vervaeke et al. 2015), this subject does not have a past medical history that is generally associated with increased implant failure risk. Nonetheless, we did not observe any dental implant failures in the placebo group during the study period.

We also used radiographic assessment to evaluate the marginal bone level around the dental implant. Peri‐implant bone loss is part of the bone remodeling process during osseointegration healing of dental implants but excessive bone loss is considered pathologic and, therefore, unfavorable. Although we did not observe any significant differences in peri‐implant bone loss between subjects in the naproxen and control groups, the variance in marginal bone loss within each group at each time point was much smaller. As a result, our study indicates that marginal bone loss, which is generally standard of care and easy to implement, is sufficient for this type of investigation.

Separately, we provided questionnaires to subjects with failed dental implants regarding their history of NSAIDs use. As expected, we found NSAID usage to be much higher in patients with a failed dental implant (67.7%) than in the general population (26.1%), as documented in the National Health And Nutrition Examination Survey (NHANES) (Davis et al. 2017). Importantly, a relatively high proportion of subjects with early implant failure in our survey did not use NSAIDs following implant surgery (32.3%). Thus, these data suggest that quantification of implant stability rather than assessing NSAID usage alone is necessary to determine the effect of these drugs on implant survival.

This pilot study has a few limitations, in addition to the small number of subjects in the trial. Due to the variation of implant sites, we were unable to standardize the implant system, diameter, and length. The shorter and narrower diameter implants are found to have less stability (ISQ), according to previous work (Barikani et al. 2013). Although the 200 mg of naproxen dosage used in this study does provide effective postoperative pain relief (Kiersch, Halladay, and Koschik 1993), 500 mg of naproxen dosage is also commonly used in clinical settings – and may influence dental implant osseointegration more strongly. The effect of rescue pain medication and antibiotics was not studied. The length of the follow‐up in this study was 4 months; however, a longer study period (e.g., 1 year or longer) may reveal that the effects of NSAIDs during initial implant osseointegration affect long‐term dental implant survival. We agree that a major limitation of the study is the sample size. Nonetheless, this data provides effect sizes and sample size estimates for future studies that can definitively determine recommendations regarding NSAID usage following dental implant surgery.

## 5 Conclusions

In conclusion, this pilot study provides effect sizes and sample size estimates for future studies to definitively determine recommendations regarding NSAID usage following dental implant surgery. Nonetheless, our study did not observe any statistically significant differences in ISQ value or marginal bone loss after up to 16 weeks of follow‐up between subjects from naproxen and placebo groups. Based on our results, we recommend future studies utilize marginal bone loss to determine the clinical impact of NSAIDs usage on dental implant osseointegration at early time points with an appropriate sample size. Furthermore, additional pilot work may be necessary to make recommendations regarding study design for the effect of NSAIDs on implants subjected to immediate loading.

## Author Contributions

*Study conception and design:* Ryan E. Tomlinson, Hattanas Kumchai, and Daniel I. Taub. *Data collection:* Ryan E. Tomlinson, Hattanas Kumchai and Daniel I. Taub. *Analysis and interpretation of results:* Ryan E. Tomlinson, Hattanas Kumchai, and Daniel I. Taub. *Draft manuscript preparation:* Ryan E. Tomlinson, Hattanas Kumchai, and Daniel I. Taub. All authors reviewed the results and approved the final version of the manuscript.

## Conflicts of Interest

The authors declare no conflicts of interest.

## Data Availability

The data that support the findings of this study are available upon request.
